# First successful pregnancies following embryo selection using Time-lapse technology in Iran: Case report

**Published:** 2015-04

**Authors:** Azita Faramarzi, Mohammad Ali Khalili, Mehrdad Soleimani

**Affiliations:** *Research and Clinical Center for Infertility, Shahid Sadoughi University of Medical Sciences, Yazd, Iran.*

**Keywords:** *Kinetics*, *Time-lapse*, *Embryo*, *Selection*

## Abstract

**Background:**

Embryo selection is a vital part of in vitro fertilization (IVF) programs, with morphology-based grading systems having been widely used for decades. Time-lapse imaging combined with embryo morph kinetics may proffer a non-invasive means for improving embryo selection. We report the first ongoing and chemical pregnancies using Time-lapse embryo scope to select best embryos for transfer in Iran.

**Cases:**

A case with tubal factor infertility was admitted to IVF program with normozoospermia. After ovarian hyper stimulation, 6 COCs were retrieved and inseminated with 25,000 progressive sperms/ oocyte. Five zygotes were placed individually into the micro wells of equilibrated embryo scope dish for Time-lapse observation, and incubated at 37°C, 5% CO2. On day 3, single embryo transfer (SET) took place based on kinetic parameters of the embryos. Clinical pregnancy was confirmed 7 weeks after SET. The second case with history of previous ICSI failure was admitted with azoospermia. Nine MII oocytes underwent ICSI, and incubated in Time-lapse facilities. The rest of procedures were followed as described for case 1. Chemical pregnancy was confirmed 15 days after SET.

**Conclusion:**

This approach opens a way to select best embryo non-invasively for SET; thus, increasing implantation, while reducing multiple pregnancy complications.

## Introduction

Many promising methods for selection of human embryos have been invented, but grading systems based on morphology criteria remained the preferred embryo assessment (1). Close relationship between morphological appearance and developmental competence of the embryo at given time points is also well documented (2). However, embryo growth is a dynamic occurrence and stationary observation of embryonic development can be limited in its ability to detect differences between embryos at same cell stages (3). By knowing the exact kinetic of development for each embryo, the selection of embryos and extended culturing could be more accurate. In addition, for conventional assessment, the embryo should be removed from the incubator; therefore, vulnerable to changes in temperature and pH of the culture medium (4). Also, there is a conflict between the need to obtain an accurate picture of embryo development and risk of disturbing stable culture conditions by use of traditional incubators. Embryo monitoring by Time-lapse use incorporated cameras in incubators which overcomes this restriction. So, it provides potential benefit of stable culture conditions during embryo survey. It also offers a promising clinical method of improved morphological evaluation to include dynamic view of the embryo (2). Here, we aim to report the first two pregnancy cases using Time-lapse embryo selections in Iran.

## Case reports

**Case 1**

A 26-year-old woman with 3 years history of tubal factor infertility was referred to Research and Clinical Center for Infertility, Yazd, Iran for in vitro fertilization (IVF) treatment. Her husband showed normal sperm analysis of 80 million/ml, 55% progressive motility, and 10% normal morphology World Health Organization (WHO). Patients were treated for ovarian stimulation routinely with long protocol. Pituitary function was suppressed using daily administration of 0.5 mg/day Intramuscular (IM) buserelin (Suprefact, Aventis, Frankfurt, Germany), started in the luteal phase of menstrual cycle. When the ultrasound showed inactive ovaries, buserelin was reduced to 0.25 mg/day IM and continued until the day of Human chorionic gonadotropin (hCG) administration. Controlled Ovarian Hyperstimulation (COH) was initiated with recombinant, Follicle-stimulating hormone (FSH) (Gonal F, Serono, Aubnne, Switzerland) 150 IU/day on the day 2 of menstrual cycle. Ovarian response was monitored by serial ultrasound examinations and evaluation of serum estradiol levels, and then gonadotropin dose adjustments were done as required. hCG (Pregnyl, Organon, Oss, The Netherlands) 10,000 IU Single dose IM was administered when at least two follicles reached a mean diameter of 18 mm. When fewer than two follicles with normal growth pattern were detected in ultrasound, the cycle was cancelled. Transvaginal oocyte retrieval was scheduled 34-36 hours after hCG administration by a 17-gauge needle (Cook, Queensland, Australia). Six cumulus oocyte complexes (COCs) were retrieved 36 h after hCG injection, and incubated in culture medium at 37°C, 6% CO2 for 3 h. The COCs were inseminated with 25,000 progressive sperms/oocyte. All 5 zygotes were placed individually into the microwells of equilibrated embryoscope dish for Time-lapse observation, and incubated until transfer. A digital Time-lapse microscope (Primo Vision, Vitrolife Company, Sweden) was used inside a CO2 incubator. The camera was set to take a picture of embryos every 5 min and the total scan of embryos was taken every 10 min. On day 3, embryos were morphologically evaluated for timing of cell divisions and development. The following early kinetic markers were assessed: time to 2nd polar body (PB) extrusion, pronuclei (PN) appearance, PN fading or syngamy (tPNf), time to 2 cells (c) (t2), 3c (t3), 4c (t4), 5c (t5), 6c (t6), 7c (t7), and 8c (t8). Durations of the second cell cycle (cc2; t3-t2) and the time to complete synchronous divisions s2 (t4-t3) were calculated. Cleavage anomaly was monitored: direct cleavage (single blastomere divided from 1 to 3 cells). Presence of multinucleation, vaculation, and fragmentation were also recorded (Table I). E4 (embryo 4) based on kinetic parameters which has shorter cleavage time was chosen for single embryo transfer (SET). The remaining embryos with longer cleavage time, uneven blastomere, fragmentation or vacuolation were cryopreserved. Clinical pregnancy was confirmed 7 weeks after SET.

**Case 2**

A 32-year-old woman and her 41-year-old husband were candidate for ICSI due to azoospermia. The hormonal profile of Luteinizing hormone (LH) =12mIU/L, FSH=5.0mIU/L, Thyroid-stimulating hormone (TSH) = 0.6uIU/L, prolactin=16ng/ml and Thyroid Peroxidase Antibody (anti TPO) = 17 IU/mL were within normal range. Testicular spermatozoa were collected by testicular sperm extraction (TESE). A total of 17 COC were retrieved, including 2 germinal vesicle (GV), 1 MI and 5 degenerated metaphase II (MII). Nine mature oocytes underwent Intra-cytoplasmic sperm injection (ICSI), and immediately incubated in Time-lapse facilities. The rest of procedures were followed as described for case 1. Table II shows morphokinetic parameters. E3 and E7 were unfertilized. E2 and E9 were discarded. E4 was best embryo based on cytokinetic parameters, with shorter timing cleavage. Thus, E4 was considered for SET. Chemical pregnancy was confirmed when the βhCG was more than180 IU/L, 14 days after embryo transfer (ET).

**Table I:** Embryos kinetics during development to day 3 (Case 1)

	**PNF**	**t2**	**t3**	**t4**	**t5**	**t6**	**t7**	**t8**	**CC2**	**S2**
E1	23:03	25:43	37:25	41:15	52:15	52:55	57:45	58:45	11:42	3:50
E2	27:03	29:23 fragment	30:43 fragment	52:15	49:05 fragment	62:08 fragment	-	-	1:20	10:52
E3	24:23	26:43 fragment	37:45 fragment	40:05 fragment decrease	52:15	44:35 vacuole	54:35	55:45	11:02	2:20
E4	23:13	24:13 fragment	26:33 uneven blastomere	52:15	37:05	40:05	46:05	49:05	02:20	9:20
E5	23:13	25:13 fragment	35:33	37:45 fragment reabsorb	47:35	48:45	48:45	49:05	10:20	02:12

E: embryo, tPNF (timing pronuclear fading), t2 (timing 2cells), t3 (timing 3cells), t4 (timing 4cells), t5 (timing 5cells), t6 (timing 6 cells), t7 (timing 7cells), t8 (timing 8cells), CC2: Second cycle cell, S2: time to complete synchronous divisions s2 (t4-t3)).

**Table II:** Embryos kinetics during development to day 3 (Case 2)

	**tESpb**	**tPNA**	**tPNF**	**tPNF**	**t2**	**t3**	**t4**	**t5**	**t6**	**t7**	**t8**	CC2	**S2**
E1	03:51	9:06	20:29	20:29	22:44	23:54	34:19	44:22	45:47 fragment	48:52 fragment	66:09 fragment	01:10	10:25
E2	03:51	08:21 vacuole	20:49	20:49	27:04	-	- Vacuole disappear	-	-	-	-	-	-
E3	2:36	-	-	-	-	-	-	-	-	-	-	-	-
E4	unfertilized	9:31	17:49	17:49	20:49	32:49	33:09 fragment	43:52 fragment	44:47 fragment	46:57 fragment	54:41 fragment	12:00	00:20
E5	1:54	8:00	28:03	28:03	32:48 Uneven blastomere	42:56	56:36	58:21	61:36	-	-	12:00	13:40
E6	1:45	7:25	26:58	26:58	29:38	40:06	40:41 uneven blastomere	54:41	60:51	-	-	12:00	00:35
E7	unfertilized	-	-	-	-	-	-	-	-	-	-	-	-
E8	1:55	6:10	23:33	23:33	26:08	26:48	39:01	49:16 fragment	50:21 fragment	54:35 fragment	58:21 fragment	00:40	12:13
E9	2:26	5:41	33:45	33:45	36:04	37:24	40:56	-	-	-	-	01:20	03:30

E: embryo, tPNF (timing pronuclear fading), t2 (timing 2cells), t3 (timing 3cells), t4 (timing 4cells), t5 (timing 5cells), t6 (timing 6cells), t7 (timing 7cells), t8 (timing 8cells), CC2: Second cycle cell, S2: time to complete synchronous divisions s2 (t4-t3)).

## Discussion

This is the first report from Iran that confirms pregnancies following embryo selection using Time-lapse technology. In present study, twelve discriminative morphokinetic parameters were identified (ESpb, PNA, PNF, t2, t3, t4, t5, t6, t7, t8, cc2, and s2) for embryos. Selected embryos for SET had shorter cleavage times compared to other embryos. In addition, they had no direct cleavage (1 cell to 3 cells), no vacuolation with smaller fragmentation during development. A relationship has been shown between early kinetics and implantation potential (3). Different dynamic parameters have been associated with embryo outcome. Predictive time-lapse markers were: early disappearance of PN, early first cleavage, and early appearance of nuclei after the first cleavage. Synchronous nuclei appearances in both blastomeres were assessed after first cleavage, t2, t3, t4, and t5 (2). In addition, Meseguer and associates showed that implantation success was strongly correlated with the timings for two time lapse markers of cc2 (or P2= time between the first and second mitosis, or the 2- to 3-cell stage) 11.9 hours, and s2 (or P3= time between or synchrony of the second and third mitosis, or the 3- to 4-cell stage) 0.76 hours. Also, strong outcome correlations exist for an additional Time-lapse marker, the time between ICSI and the 5-cell embryo stage (5). We recommend well-designed randomized clinical trials are needed to survey the most important dynamic parameter associated with embryo outcome.

Despite efforts to optimize embryo selection methods, there is relatively low IVF success with a clinical pregnancy rate of~30% per transfer (2). This often results to the transfer of more than one embryo at a time, which increases the risk of multiple pregnancies associated with neonatal and maternal complications (6). Efficient method to reduce the risk of multiple pregnancies is elective SET. There have been studies in search of viability markers to complement common criteria for selection, e.g aneuploidy screening, O2 respiration, metabolic profiling and gene expression analysis (7). Although, many embryo selection procedures are promising, morphology grading system is the preferable way of evaluating embryo development. Compared to alternative methods, static morphological grading is used greatly, because of its simplicity and cost-effectiveness (8). Furthermore, with the conventional embryo assessment outside the incubator, embryo was exposed to variations of temperature and pH of culture medium. Thus, a challenge exists between the necessity to obtain a detailed picture of embryo growth and risk of disturbing stable culture condition (2). Time-lapse imaging is an emerging instrument which let to identify parameters that can foretell the developmental potential of cleaving embryos noninvasively (9, 10). Use of Time-lapse caused constant and fully controlled culture conditions; while, handling of embryos outside the incubator are minimized (11). When using Time-lapse, there is no need to frequently remove the embryos from the incubator for assessment therefore the doors is opened less repeatedly. In addition uninterrupted embryo evaluation significantly diminishes manual handling embryos by Time-lapse, leaving the embryos under stable development conditions. In addition, risk of contamination within the laboratory is reduced (12). Time-lapse gives extra information about embryo growth to embryologists; this data may be used to better our ability to select embryos for SET. Continuing monitoring with use of Time-lapse lets a more exact identity of embryos that follow likely chromosomally normal (5, 12). Although, Park *et al* showed no differences in embryo quality when comparing culture in a standard incubator with that of a closed Time-lapse imaging incubator. They noted that in spite of the less improvement of embryo development, the whole developmental process could be documented in Time-laps facility. Also, significant events could be assessed retrospectively at any time before embryo selection for transfer (13).

## Conclusion

In conclusion, this first report from Iran showed optimism regarding the successful application of Time-lapse technology for SET in assisted reproduction program.

## Conflict of interest

There is no conflict of interests of each author.
